# Electrochemical Properties of Screen-Printed Carbon Nano-Onion Electrodes

**DOI:** 10.3390/molecules25173884

**Published:** 2020-08-26

**Authors:** Loanda R. Cumba, Adalberto Camisasca, Silvia Giordani, Robert J. Forster

**Affiliations:** 1 School of Chemical Sciences, National Centre for Sensor Research, Dublin City University, Dublin 9, Ireland; Adalberto.camisasca@dcu.ie (A.C.); robert.forster@dcu.ie (R.J.F.); 2 FutureNeuro SFI Research Centre, Dublin, Ireland

**Keywords:** carbon nano-onion, ink formulation, screen-printed electrode, carbon nanomaterial, dopamine

## Abstract

The properties of carbon nano-onions (CNOs) make them attractive electrode materials/additives for the development of low-cost, simple to use and highly sensitive Screen Printed Electrodes (SPEs). Here, we report the development of the first CNO-based ink for the fabrication of low-cost and disposable electrodes, leading to high-performance sensors. Achieving a true dispersion of CNOs is intrinsically challenging and a key aspect of the ink formulation. The screen-printing ink formulation is achieved by carefully selecting and optimising the conductive materials (graphite (GRT) and CNOs), the polymer binder, the organic solvent and the plasticiser. Our CNO/GRT-based screen-printed electrodes consist of an interconnected network of conducting carbon particles with a uniform distribution. Electrochemical studies show a heterogeneous electron transfer rate constant of 1.3 ± 0.7 × 10^−3^ cm·s^−1^ and a higher current density than the ferrocene/ferrocenium coupled to a commercial graphite SPEs. In addition, the CNO/GRT SPE can detect dopamine in the concentration range of 10.0–99.9 µM with a limit of detection of 0.92 µM (N = 3). They exhibit a higher analytical sensitivity than the commercial graphite-based SPE, with a 4-fold improvement observed. These results open up the possibility of using high-performing CNO-based SPEs for electrochemical applications including sensors, battery electrodes and electrocatalysis.

## 1. Introduction

Screen-printed electrodes (SPEs) represent a leading technology for the mass production of inexpensive, highly reproducible and disposable electrochemical sensing platforms [1,2]. They are highly versatile and can be designed in many configurations, such as interdigitated and independently addressable arrays, as well as in different electrode size and thickness. However, the ink composition, for example, material type, size and loading, critically influences the overall performance of the electrodes [1].

Thus, it is highly important to formulate novel inks so that the properties of the resulting electrodes, such as the microstructure and surface chemistry, can be predictably controlled and optimised [3].

Different nanomaterials have been employed for the fabrication of SPEs [4]. The ability to modify and adjust the electrode composition to deliver particular properties, while maintaining the process cost-effectiveness, allows SPEs to be produced with high flexibility and selectivity to precise analytical targets [5]. In addition, through the careful choice of the nanomaterials used, the overall electrode performance can be greatly enhanced in terms of sensitivity, detection limit and stability [6].

Carbon-based inks, consisting of graphite and/or carbon particles mixed with polymer binders, solvents and additives, are an appealing strategy for SPE fabrication due to their low cost, broad potential windows and relatively low background currents [1,5].

Carbon nanomaterials (CNMs) are very attractive for the mass production of SPEs and represent a significant opportunity to enhance the analytical sensitivity of these devices [7], enabling new sensing applications such as the early detection of disease through enhanced sensitivity [3,8,9]. Among them, carbon nanotubes (CNTs) [10,11] and graphene-related materials [12] have proven to be efficient electrode materials as they exhibit remarkable chemical, mechanical and electronic properties, high surface areas, as well as low cost. Additionally, the ability to functionalise their surfaces with antibodies, nucleic acids or catalysts, can lead to enhanced analytical performance, including sensitivity and selectivity [13,14,15].

In this contribution, we report on screen printed electrodes that incorporate carbon nano-onions (CNOs); a member of the carbon family that consists of concentrically nested spherical fullerenes [16]. Compared to other CNMs such as graphene, CNO synthesis is highly appealing from an industrial point of view.

Previous reports [17,18] have demonstrated that the properties of carbon nanomaterials can be significantly affected by their structure, including defects. The ability to produce CNOs via thermal annealing of detonation nanodiamonds [19,20] represents a useful additional technique for producing carbon-based nanomaterials that are nanoparticle in character, size monodisperse and highly pure.

In the last decade, the biological [19,21] and electrochemical [22,23] applications of CNOs have significantly expanded due to the favourable properties of the nanomaterial; including their small size, large accessible surface area, high biocompatibility, as well as outstanding physico-chemical properties [24]. Furthermore, in terms of using CNOs as the electrode material, additional features such as high conductivity, chemical stability as well as ease surface modification, make this material very attractive for biosensing applications.

In this regard, recent studies have demonstrated that electrodes coated with CNOs [25,26,27] or CNOs embedded within a polymer coating [28,29] deliver higher sensitivity and lower limits of detection compared to standard glassy carbon electrodes. For example, Ibanez-Redin and co-workers reported on the detection of the pancreatic cancer biomarker CA19-9 using an SPE, which was modified with layers of both CNOs and graphene oxide (GO). The addition of CNOs resulted in higher sensitivity and a lower detection limit compared to graphene oxide alone, suggesting that CNOs can enhance the overall biosensor performance [30]. However, surface coating methods onto expensive substrates can result in the heterogeneous distribution of the nanomaterials onto the electrode surface, thus leading to poor reproducibility and, most importantly, a lack of long-term stability.

In this contribution, we report the first CNO-based ink formulation for the production of high-performance screen-printed electrodes (CNO/GRT SPEs). The ink formulation was optimised by varying the loading of CNOs and graphitic materials as well as the additives/binders, so as to maximise the content of the conducting species and to minimise CNO aggregation. The chemical composition and morphology of the CNO/GRT SPEs were characterised using scanning electron microscopy (SEM), energy-dispersive X-ray spectroscopy (EDS), X-ray photoelectron spectroscopy (XPS) and Raman spectroscopy. Furthermore, their electrochemical performance was evaluated by cyclic voltammetry and compared to commercial graphite-based SPEs (GRT SPEs). In particular, we demonstrate that, compared to a commercial GRT SPE, the CNO/GRT SPE has a higher analytical sensitivity and exhibits an enhanced electrochemical performance for the detection of dopamine.

## 2. Results and Discussion

Similar to other CNMs, pristine CNOs are highly hydrophobic and tend not to disperse well in organic and aqueous solvents due to aggregation, which can limit practical applications [24,31].

**Figure 1:**
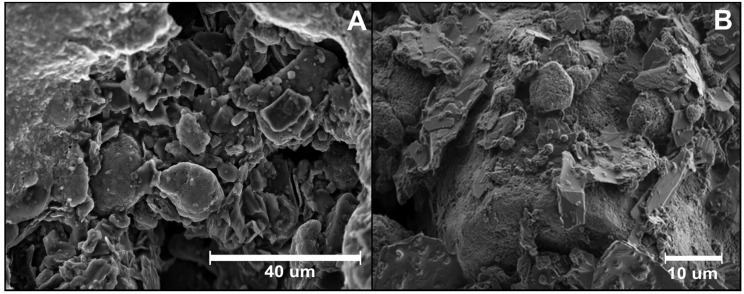
SEM images of the CNO/GRT SPE surface at 10 kV as accelerating voltage and different magnifications. (**A**) 1300×; (**B**) 2000×.

Despite being stabilised with di(propylene glycol) monomethyl ether, the CNOs used here show a strong tendency to aggregate in a wide range of solvents. The ink composition was optimised so as to avoid any additional aggregation but SEM suggests that the CNOs exist as aggregates with dimensions of up to a few µm (Figure 1).

For the ink formulation, we performed a careful selection and content optimisation of the materials. The content of each ink component used in the final optimised ink formulation is reported in Table 1. The working electrode mainly consists of CNOs with the addition of a small percentage of graphite in order to obtain a highly conductive ink.

We exploited the percolation theory to help identify the optimal nanomaterial content. The percolation theory describes the transition from an insulating to a conductive material in terms of percentage of conductive particles added. Insulating polymers can exhibit a significant increase in electrical conductivity at a critical particle loading, namely the percolation threshold [32]. The percolation threshold strongly depends on the aspect (length-to-diameter) ratio of the conductive particles [33,34]. In our ink formulation, a 31 wt.% of conductive particles was required to reach the percolation threshold.

A polymeric binder, polyhydroxyether, was used due to its mechanical properties, ability to bind conductive particles across a wide range of loadings, as well as its dielectric properties. Di(propylene glycol) methyl ether was selected as the organic solvent as it gives a homogeneous dispersion of the carbon materials. In addition, it provides high stability to the ink formulation during the printing process while being less volatile than other organic solvents with similar properties. The plasticizer was poly(dimethylsiloxane-co-methylphenylsiloxane) and polyethylene terephthalate (PET) was used as a flexible plastic substrate and was pre-treated to enhance the adhesion of the ink.

In order to find the best ink formulation in terms of CNM contents, we systematically varied the CNO/GRT% ratios. Inks containing 70, 60 and 50 wt.% of CNO particles (relative percentage of other materials kept constant) were tested. The cyclic voltammograms of the different CNO/GRT SPEs, shown in Figure S1 (Supplementary materials), were recorded in 1 mM FcMeOH/PBS pH 7.4 at 50 mV·s^−1^ (vs. pseudo Ag/AgCl). By decreasing the weight percentage of the CNO particles on the ink formulation, it was possible to observe a significant increase in the peak-to-peak separation, going towards an irreversible process until no redox peaks are observed. For this reason, only SPEs based on the optimised ink are discussed.

After the curing process, the optimised CNO/GRT SPE ohmic resistance was measured. The overall electrode resistance could be associated to the bulk resistance of fillers, constriction resistance (i.e., sites where there is direct contact between the conductive fillers) and tunnelling resistance. Tunnelling resistance comes from sites where conductive particles are not directly connected and electrons must overcome energy barriers to transfer between the particles [35,36]. The resistance strongly influences the electrochemical performance at the electrode interface and, for some formulations, makes the electrodes unsuitable for voltammetric applications. The SPEs with the optimised CNO/GRT loading have an ohmic resistance of 800 ± 24 Ω·cm^−1^ (n = 20), making them suitable for voltammetry.

### 2.1. Structural and Chemical Properties

The CNO/GRT SPEs were characterised by SEM, EDS, XPS and Raman spectroscopy to probe the morphological and chemical composition of the electrodes.

Figure 1 depicts typical SEM images of the electrode at different magnifications. In the lower magnification image (Figure 1A), the electrode surface reveals the presence of a uniform and highly interconnected carbon network distributed throughout the polymer matrix, creating a high surface area.

The higher magnification image, reported in Figure 1B, shows a microstructure consisting of irregularly shaped flakes of graphite with size ranging from a few to tens of µm and spherical aggregates, ascribed to the presence of carbon nano-onions, with dimensions from hundreds of nm up to 5 µm. Figure S2 (Supplementary materials) provides further evidence of the different shape of the two carbon materials.

Provided that the polymeric binder does not insulate the particles, the rough nature of the electrode surface should ensure a larger real active area than the geometrical one, which can enhance the analytical response.

### 2.2. Elemental Composition

Table 2 reports the EDS results and the theoretical chemical composition based on the materials employed for printing the electrodes; note that the calculation has been made excluding any contribution from residual organic solvent or contributions from the substrate material.

Besides the carbon (C) and oxygen (O) signals arising from the use of the graphitic materials and the additives (polymeric binder and plasticiser), the additional presence of silicon (Si) on the electrode surface is attributed to the poly(dimethylsiloxane-co-methylphenylsiloxane) used in the ink formulation as plasticiser.

From the analysis of the experimental results, we can observe that the Si content is in perfect agreement with the theoretical composition, while differences can be observed for C and O, with a much higher oxygen content obtained from the EDS analysis. Despite being a semi-quantitative technique, this mismatch can be tentatively attributed to residual traces of organic solvent in the electrodes after the curing process and to the contribution of the PET substrate.

**Figure 2:**
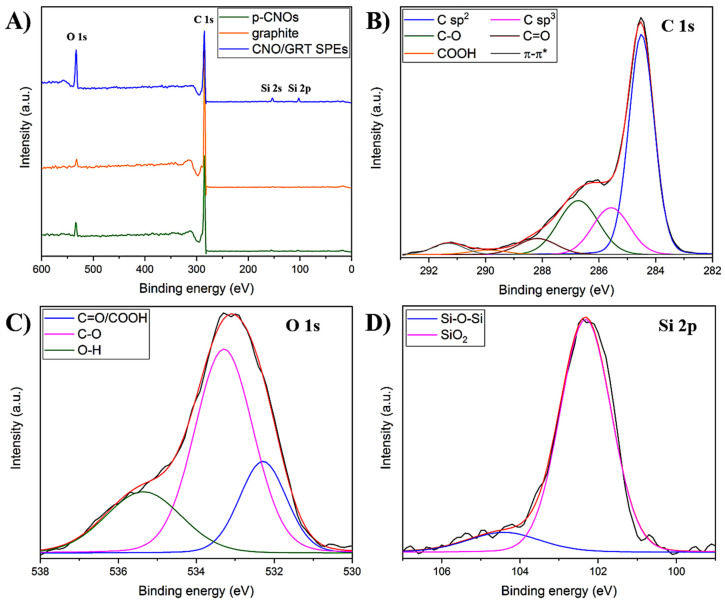
(**A**) XPS survey spectra of p-CNOs, graphite and CNO/GRT SPEs; high-resolution C 1s (**B**), O 1s (**C**) and Si 2p (**D**) XPS spectra of CNO/GRT SPEs, including peak deconvolution. The experimental and fitting curves are shown in black and red, respectively.

XPS analyses were performed to further investigate the chemical composition of these materials. Figure 2A reports the XPS survey spectra of the pristine CNMs employed for the electrode fabrication and the CNO/GRT SPE; their elemental composition is shown in Table 3.

The pristine CNOs and graphite exhibit a high content of C along with a small percentage of O (5.6 and 2.9, respectively), while the negligible contribution of Si (<1% in both cases) is attributed to the silicon substrate used for the analyses. The composition of the CNO/GRT SPE is indistinguishable from that obtained from EDS (Table 2).

The chemical state of the different elements was investigated using high-resolution XPS spectra and the results are reported in Tables S1 and S2 (Supplementary Materials). The C 1s spectra of p-CNOs and graphite (Figure S3 in Supplementary Materials) can be deconvoluted into six components. The most intense peak, centred at 284.5 eV is assigned to sp^2^ carbon atoms, while the other contributions are attributed to carbon atoms with sp^3^ hybridisation, single and double oxygen-bonded carbon species (mainly C–O and C=O and a negligible presence of COOH) as well as a π–π* transition peak [21,23]. In particular, the analysis of Table S1 suggests that p-CNOs consist of a more defective structure than that of the graphite. Similar contributions can be observed for the CNO/GRT SPEs (Figure 2B) but with a much higher content of C–O species, which is attributed to the presence of the additional oxygen-containing groups in the binder.

Deconvolution of the O 1s peak of p-CNOs and graphite (Figure S4 in Supplementary Materials) shows the presence of two different contributions. The first peak, located at around 532 eV, is due to mixed C=O/COOH states, while the peak at higher binding energy is assigned to the presence of C–O species [37,38,39]. The analysis of the O 1s spectrum of CNO/GRT SPE, reported in Figure 2C, reveals an additional peak at approximately 535 eV, which can be assigned either to the presence of chemisorbed O-H groups, resulting from adsorbed water onto the electrode surface or oxygen in SiO_2_ [40,41]. Also, the peak at 533.3 eV includes the contribution of oxygen atoms belonging to siloxane groups [42]. From the analysis of Table S2, it can be inferred that the relative percentages of the different contributions in the O 1s peak are in good agreement with those reported in the C 1s (Table S1).

To better understand the origin of the Si signal, a high-resolution XPS spectrum of Si 2p core level of the CNO/GRT SPE was acquired (Figure 2D). After peak fitting, two different contributions can be observed (Table S3 in Supplementary Materials). The most intense peak at 102.3 eV corresponds to Si atoms in the siloxane (Si–O–Si) and Si-C groups present in the chemical structure of the plasticiser [42,43,44], while the peak at 104.4 eV is assigned to the native oxide layer (SiO_2_) of the Si substrate employed for the analyses [41,45].

### 2.3. Raman Spectroscopy

**Figure 3:**
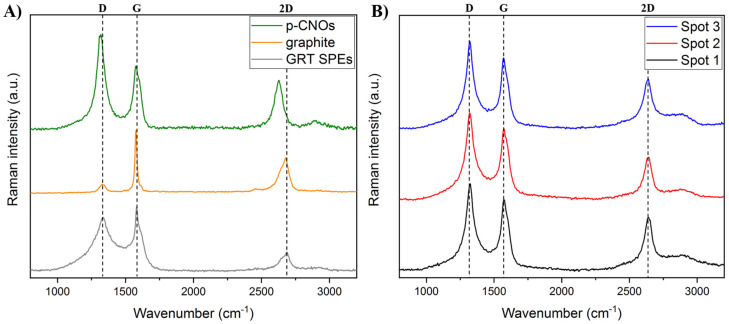
Raman spectra of (**A**) p-CNOs (green), graphite (orange) and GRT SPEs (grey) and (**B**) CNO/GRT SPEs at three different spots. A 633 nm laser source and a laser power density below 10^5^ W/cm^2^ were used for the analyses. D-, G- and 2D-bands position are shown in the spectra.

Electrochemistry is intrinsically an interfacial process and Raman spectroscopy represents a powerful, non-destructive approach to probing the interfacial chemical composition; such as the presence of CNOs at the electrode surface. The Raman spectra of p-CNOs, graphite, GRT SPEs and CNO/GRT SPEs are reported in Figure 3. They reveal the typical Raman features associated with carbon nanostructures, consisting of two main peaks in the 1300–1600 cm^−1^ range. The D-band, located at around 1320 cm^−1^, arises from the presence of defects in the graphitic skeleton, while the G-band, centred at approximately 1580 cm^−1^, is associated with the E_2g_ vibration mode of sp^2^-hybridized carbon atoms. Furthermore, the additional feature at higher wavenumbers, namely the 2D-band, corresponds to the overtone of the D-band and is due to a second-order Raman scattering [46,47,48].

The Raman spectra of p-CNOs, graphite and GRT SPEs, shown in Figure 3A, exhibit similar features but notable differences in terms of peak positions and intensities. The spectrum of graphite is dominated by two intense peaks. A typical narrow G-band and a broad asymmetric 2D-band can be observed at 1577 and 2677 cm^−1^, respectively, along with a small intensity D-band at 1328 cm^−1^ [49,50]. The GRT SPE shows similar features, with a more pronounced D-band, suggesting that the SPE fabrication introduces some additional defects. This is further corroborated by the appearance of the disorder-induced D’-band at around 1600 cm^−1^ and the increase in the I_D_/I_G_ ratio (from 0.16 for graphite to 0.84 for the SPE), which is commonly employed to give information about the disorder present in graphitic materials [50].

Compared to graphite, p-CNOs exhibit a very prominent D-band with respect to the G-band with a I_D_/I_G_ ratio of 1.49 and the additional presence of a weak defect-activated Raman feature at around 2900 cm^−1^, known as the D+G-band, which is associated with combination scattering. These results are in agreement with the high defect density reported for CNOs synthesised by thermal annealing [21,23] and in line with the XPS results. Furthermore, a downshift in the G- (~2 cm^−1^), D-(~10 cm^−1^) and 2D-band (~50 cm^−1^) frequencies compared to those of graphite is observed, which is attributed to the tensile strain introduced, during the heat treatment, by the curvature of the graphitic planes [51,52].

Figure 3B shows the Raman spectra of the CNO/GRT SPEs at three different spots, showing similar peak positions and I_D_/I_G_ ratio values (i.e., 1.23) for each investigated spot. Furthermore, the Raman spectra resemble that of p-CNOs, as expected from their high content in the ink, exhibiting the D-, G- and 2D-bands at 1321, 1571 and 2640 cm^−1^, respectively. The slightly lower I_D_/I_G_ ratio compared to that of p-CNOs (1.23 vs. 1.49) can be attributed to the presence of graphite, which enhances the graphitic character of the carbon mixture.

Our results confirm the presence of the carbon materials over the electrode surface and suggest that the graphitic materials are uniformly distributed within the surface of the SPE, as similar spectra are observed across different areas.

### 2.4. Electrochemical Properties

The electrochemical behaviour of the CNO/GRT SPEs has been investigated by cyclic voltammetry. One key impact of including 2D and 3D carbon nanomaterials within screen-printed electrodes is that their electrochemical properties, such as potential window, rate of electron transfer and double layer capacitance, may differ from traditional graphite-based systems.

**Figure 4:**
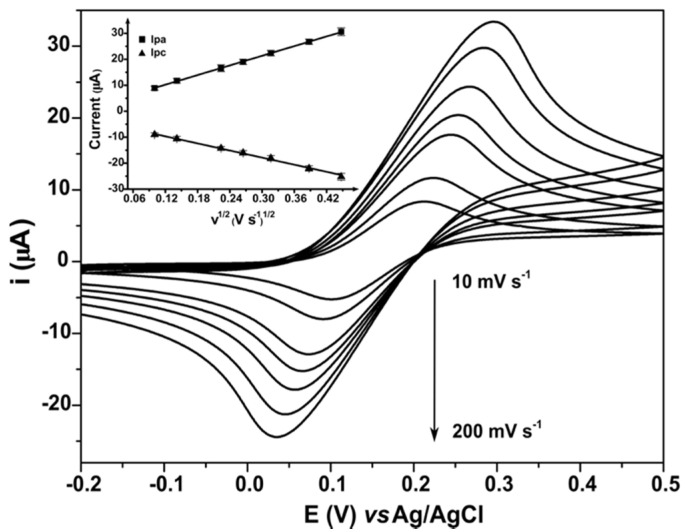
Cyclic voltammograms of the CNO/GRT SPE in 1 mM FcMeOH/PBS pH 7.4 at different scan rates (from bottom to top 10, 20, 50, 70, 100, 150 and 200 mV·s^−1^). Inset: I_pa_ and I_pc_ versus square root of scan rate (υ^1/2^).

Figure 4 and Figure S5 shows cyclic voltammograms of the CNO/GRT and commercial SPEs, respectively, for 1 mM FcMeOH in PBS at pH 7.4 as the supporting electrolyte. The scan rate was systematically varied from 10 to 200 mV·s^−1^, without ohmic drop compensation.

A well-defined peak corresponding to the Fc/Fc^+^ is observed at an apparent formal potential (E^0′^) of approximately +0.15 V for CNO/GRT SPE and +0.28 V for the commercial SPE at 50 mV·s^−1^. This difference in apparent formal potentials is observed even at low scan rates where the rate of heterogeneous electron transfer does not influence the behaviour. This suggests that the free energy for oxidation at the CNO/GRT surface is approximately 12.5 kJ·mol^−1^ lower than the graphite surface. This is most likely due to differences between the two interfaces, for example, surface charge, type and coverage of binding/electron transfer mediating functional groups, hydrophobicity and so forth.

The insets in Figure 4 and Figure S5 show a linear relationship between the peak current, I_pa_ and I_pc_ and the square root of scan rate, υ^1/2^, indicating that semi-infinite linear diffusion dominates the response at both electrodes. Furthermore, the ratio of the anodic and cathodic peak currents is unity within experimental error, indicating that the response is chemically reversible.

In addition, as shown in Figure 4 and Figure S5, the polarisation behaviour is slightly accentuated for the CNO/GRT SPE when compared to the commercial SPE.

**Figure 5:**
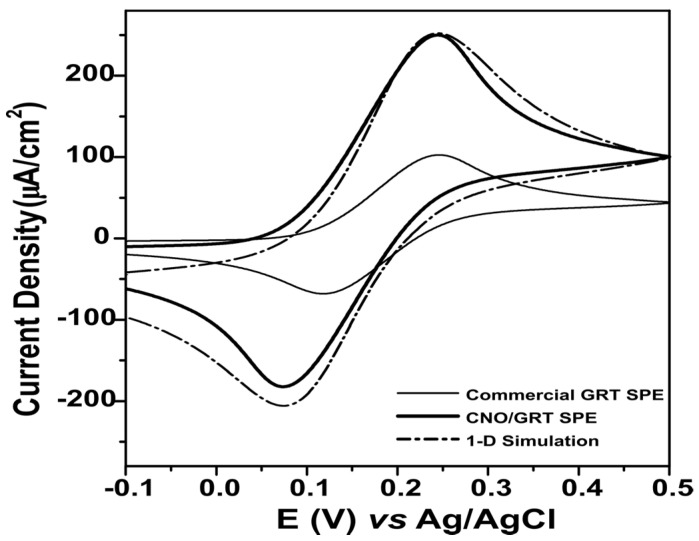
Comparison of the electrochemical behaviour using cyclic voltammetry of the CNO/GRT SPE, commercial GRT SPE and CNO/GRT SPE 1D simulation in 1 mM FcMeOH/PBS pH 7.4 at 50 mV·s^−1^ (vs. pseudo Ag/AgCl). Simulation parameters: alpha = 0.48, E^0′^ = +0.15 V, D_ox_ = 7.5 × 10^−6^ cm^2^·s^−1^, D_red_ = 9.9 × 10^−6^ cm^2^·s^−1^ and v = 50 mV·s^−1^).

The influence of the scan rate on the anodic and cathodic redox peak potentials allows the heterogeneous electron transfer rate constant, k^0^, to be determined. A large k^0^ is highly desirable so that the response is reversible even at fast scan rates. For the CNO/GRT SPEs, increasing the scan rate from 10 to 200 mV·s^−1^ causes the peak-to-peak separation (∆E_p_) to increase from 110 to 260 mV, indicating that the time constant for electron transfer becomes comparable to the experimental time over this range. Figure 5 shows that a lower ∆Ep is observed for the graphite-based SPE. There are several factors that could lead to this behaviour, such as the intrinsic resistance of the complete electrode assembly. Specifically, unlike the commercial graphite-based SPE, the track linking of the CNO/GRT working electrode to the external connector does not have a highly conducting silver layer underneath the carbon surface. A silver layer decreases the overall electrode resistance and therefore decreases the peak-to-peak separation observed, especially at higher scan rates where the current is larger. However, while a silver underlayer does not present any difficulty for rapid, single-shot measurements, for example, glucose monitoring [53], it can contaminate both the electrode and sample at longer times and substantially increases the manufacturing costs.

The heterogeneous electron transfer rate constants for the CNO/GRT and the commercial GRT SPEs were determined using a 1D simulation of the full CV and using the standard Nicholson-Shain approach that uses only the shift in peak potentials with scan rate [54]. Both approaches gave indistinguishable values for the standard heterogeneous electron transfer rate constant. The k^o^ values obtained for ferrocene at the CNO/GRT and GRT electrodes were 1.3 ± 0.7 × 10^−3^ and 4.0 ± 0.9 × 10^−3^ cm·s^−1^, respectively. This lower electron transfer rate for the CNO/GRT SPEs may be attributed to the higher percentage of polymer binder necessary to give a good ink adhesion on the plastic substrate, since electron transfer across this material could decrease k^o^ [55].

Figure 5 compares the electrochemical behaviour of the CNO/GRT and commercial GRT SPEs. These data suggest that both electrodes generally present similar electrochemical, quasi-reversible, behaviour for the Fc/Fc^+^ couple. However, despite the closely matched geometric areas, for both oxidation and reduction, the faradaic currents observed for the CNO/GRT SPEs are larger than the commercial GRT SPEs. Since the k^o^ values are very similar, the difference is not explained by heterogeneous electron transfer dynamics. The depletion layer thickness is approximately 20 µm (50 mV·s^−1^) for CNO/GRT SPE and will be thinner for higher scan rates. As shown in Figure 1, the CNO SPE has aggregates and pores that have a comparable or larger length scale. Under these conditions, the area available for electron transfer will be larger than the geometric area resulting in higher currents [56].

There are more substantial differences in behaviour between the two types of electrode for ferrocenium reduction where the cathodic peak potential (E_pc_) is approximately 50 mV more negative for the CNO/GRT than the GRT SPE. This result indicates that it is thermodynamically more difficult to reduce the oxidised ferrocenium cation at the CNO based electrode, most likely due to electrostatic repulsion of the ferrocenium cation by positive charges with the CNO electrode creating an additional activation overpotential.

### 2.5. Biomolecule Detection

Dopamine (DA) is crucial to the maintenance of physiological processes, including cognition, motor activity, memory and emotional regulations [57]. However, the unbalanced activity of this neurotransmitter may lead to dysfunctions related to neurodegenerative diseases [58]. The electrochemical detection of dopamine is challenging, for example, it is difficult to get a well-defined oxidation response and its electrochemically irreversible behavior often leads to electrode fouling.

**Figure 6:**
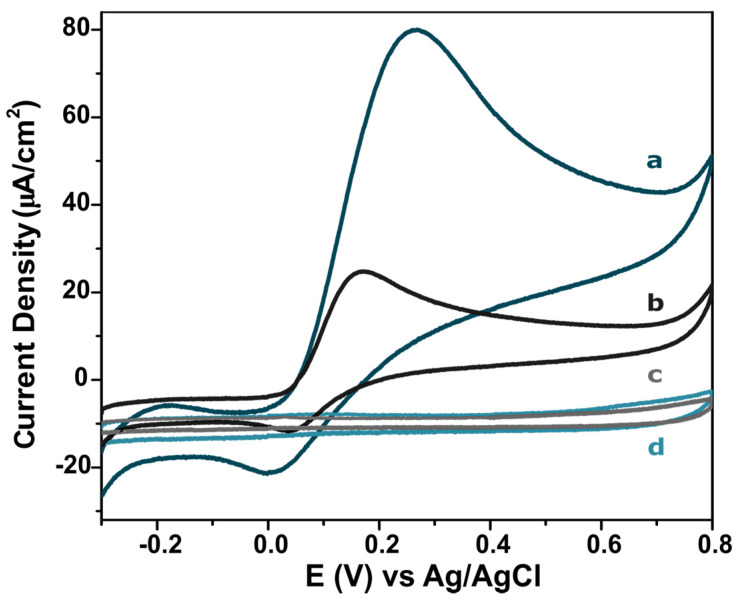
Cyclic Voltammograms of (**a**) CNO/GRT SPE and (**b**) commercial SPE in the presence of 99.9 μM DA in PBS pH 7.4, (**c**) CNO/GRT SPE and (**d**) commercial SPE in the absence of DA (only in PBS pH 7.4). Scan rate: 50 mV·s^−1^ (vs. pseudo Ag/AgCl).

Figure 6a,b show that a well-defined oxidation peak corresponding to the dopamine/dopamine quinone is observed at +0.26 V and +0.18 V (vs. pseudo Ag/AgCl) at 50 mV·s^−1^ for the CNO/GRT and commercial SPEs, respectively. The CVs exhibit a quasi-reversible behavior. An important issue in the voltammetric behavior is the capacitive current that is highly influenced by the specific surface area. We have used the electrode area determined at high scan rate, where the depletion layer is thin and the response most sensitive to the surface roughness, to determine the specific interfacial capacitance as 42 ± 1.8 µF·cm^−2^ for both electrodes. This value is consistent with that expected for a pristine carbon surface. Significantly, the ratio of the capacitance to faradaic current is approximately 1:15, allowing the current associated with the DA oxidation to be accurately recorded. In the absence of DA, no specific redox peaks were observed, as shown in Figure 6c,d.

**Figure 7:**
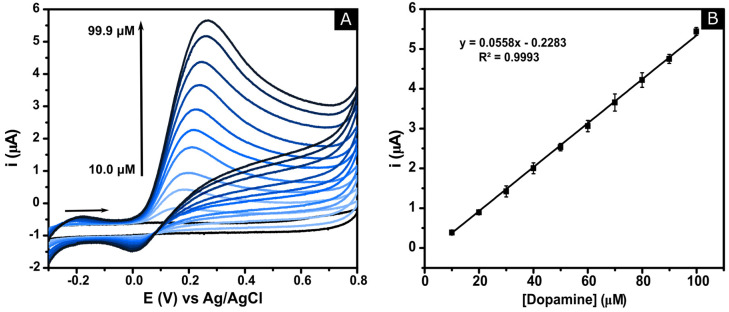
(**A**) Cyclic voltammograms of CNO/GRT SPE in PBS pH 7.4 after subsequent additions of DA, in a range of 10.0 to 99.9 µM. (**B**) Calibration plot of the anodic peak current as a function of the DA concentration (N = 3). Scan rate: 50 mV·s^−1^.

Subsequently, additions of DA were made into 10 mM phosphate buffer solution (pH 7.4) over the range of 10.0 to 99.9 μM. As depicted in Figure 7A (CNO/GRT SPE) and S6A (commercial GRT SPE), the increase in the current intensity of the oxidation peak is directly proportional to the concentration of DA in solution. There was also an anodic shift in the onset potential of +0.08 V for the CNO/GRT SPE and +0.06 V for the commercial SPE.

Figure 7B and Figure S6B show the calibration curves for the CNO/GRT and commercial GRT SPEs for DA. Both analytical responses presented a linear relationship between the DA concentration and peak current with regression equations of y = 0.0558x − 0.2283 (R^2^ = 0.9993, N = 3) and y = 0.0371x − 0.0812 (R^2^ = 0.9971, N = 3), respectively. The limit of detection (LOD, based on 3σ/slope) for the CNO/GRT SPE was found to be 0.92 µM and for the commercial SPE 3.41 µM. The analytical sensitivity (slope of the calibration curve) is approximately 4 times higher for the CNO/GRT SPE compared to the commercial GRT SPE for dopamine. This improved performance may be associated with the oxygenated species on the electrode surface due to the incorporation of the CNO particles in the ink formulation, which facilitates electrocatalytic reactions [59].

The percentage relative standard deviation (%RSD) can be seen on the error bars of Figure 7B and Figure S6B with values of 2.7 and 5.2% RSD for CNO/GRT and commercial SPEs, respectively. The low %RSD confirms that the CNO/GRT SPE manufacturing process is highly stable and reproducible.

Table 4 compares the analytical performance of the CNO/GRT SPE to different previously reported electrode platforms modified with carbon (nano)materials for the electrochemical detection of DA. While many of these previous reports use differential or square wave voltammetry to enhance the detection sensitivity, the CNO/GRT SPE LOD and dynamic range compare favourably suggesting it may be a useful platform for the development of low-cost sensors for biologically relevant molecules.

## 3. Materials and Methods

### 3.1. Materials

Detonation nanodiamonds (DNDs) were purchased from Carbodeon Ltd. (Vantaa, Finland). The synthesis of pristine CNOs (average particle size 6 nm) was performed by thermal annealing of DNDs following a previously published methodology [21,23]. All chemicals were purchased from Sigma-Aldrich (Wicklow, Ireland), Wacker Chemicals Ltd. (Munich, Germany), Cabot (Riga, Latvia), Imerys Graphite & Carbon (Bironico, Switzerland) and Alfa Aesar (Lancashire, UK) (highest purity grade available; 97 to 99.9% purity). All reactions and measurements were carried out under ambient conditions, unless otherwise specified. The solutions were used on the same day of preparation after being purged with nitrogen gas for 15 min prior to electrochemical analysis. Solutions at lower concentrations were prepared by appropriate dilution with Ultrapure MilliQ water (resistivity 18 MΩ·cm^−1^) of a concentrated stock solution of ferrocenemethanol (FeMcOH), dopamine (DA) hydrochloride, phosphate buffer (PBS; pH 7.4) and sulfuric acid (H_2_SO_4_).

### 3.2. Instrumentation

SEM images were obtained with a Hitachi S5500 Field Emission SEM (Hitachi, Krefeld, Germany) equipped with energy-dispersive X-ray spectroscopy (EDS) operated at an acceleration voltage of 10 kV.

XPS analyses were performed using a Scienta Omicron ESCA 2SR XPS system (Scienta Omicron, Uppsala, Sweden) equipped with a monochromatic Al Kα (hν = 1486.6 eV) X-ray source. For the analyses, the samples were loaded onto carbon adhesive pads located on a thin Silicon wafer. Survey spectra were acquired with 100 eV pass energy and 1.0 eV step size, while high-resolution spectra with 50 eV pass energy and 0.1 eV step size. Data analysis was carried out using Multipak 9.6 software and the XPS spectra were calibrated to the C 1s peak at 284.5 eV.

Raman spectra were acquired at room temperature by using a Renishaw 1000 micro-Raman system (Renishaw, New Mills, UK) equipped with a Leica microscope with a 50× magnification objective and a 633 nm HeNe laser source. To avoid sample heating, the laser power density was kept below 10^5^ W/cm^2^. All spectra were baseline-corrected and normalised with respect to the G-band.

Voltammetric measurements were performed using a CH Instrument (760e) potentiostat (CH Instruments, Austin, TX, USA). The electrodes were used as printed and were not pre-treated before the electrochemical measurements. All analysis were performed at room temperature. A three-SPE system was applied to all measurements-CNO/GRT and GRT as working electrodes (3 mm diameter), pseudo Ag/AgCl as a reference electrode and carbon as the counter electrode. All the electrical connections were printed using a carbon ink, except for the CNO/GRT, which was printed with the same material of the working area. Silver ink was not applied underneath the carbon electrical connections. The DA concentrations analysed were 10.0, 20.0, 30.0, 40.0, 50.0, 60.0, 70.0, 79.9, 89.9 and 99.9 µM. The commercial GRT SPE tested are from Metrohm DropSens (DRP-11L-4 mm diameter, supplied by Metrohm, Runcorn, UK) with an ohmic resistance of 432 ± 38 Ω·cm^−1^ (n = 20).

The electrochemical computational modelling was performed using KISSA-1D; software version 1.2; for simulation of electrochemical reaction mechanisms. The resistance of the electrodes was measured with a Keithley 2420-C 60V/3A/60W SourceMeter-2-point probe (Keithley Instruments, Solon, OH, USA). The probes were spaced at 24.9 mm along the tracks of the SPEs.

### 3.3. CNO/GRT Optimised Ink Formulation

First, the polymeric binder, polyhydroxyethers (10 wt.%), was dissolved in an organic solvent, di(propylene glycol) methyl ether (55 wt.%) and then the plasticizer (4 wt.%) poly(dimethylsiloxane-co-methylphenylsiloxane) was incorporated into the mixture. Thereafter, a high load of carbon nano-onions (24 wt.%) and a small portion of graphite (GRT) (7 wt.%) were added to the mixture. All powder materials (conductive particles) were mixed into the viscous liquid using a high-shear PRO250 homogeniser. The mixture was blended for 20 min at 10,000 rpm for the first 5 min, then gradually increased to 20,000 rpm for the next 15 min and then left stirring at 1500 rpm for 2 h at 50 °C and subsequently stored at room temperature.

### 3.4. Substrate Surface Pretreatment

The polyethylene terephthalate (PET) substrates were pre-treated prior to electrode printing to modify the surface energy and increase adhesion & stability of the CNO/GRT ink on the substrate. First, the substrate was cleaned with ethanol and immersed in sulfuric acid (1.0 M, aqueous) for 5 min and sodium hydroxide (0.5 M, aqueous) for 3 min. After several washes with Milli-Q water, the treated PET was finally rinsed with ethyl triglycol (ink primer).

### 3.5. CNO/GRT SPE Printing Process

Each of the three electrodes; CNO/GRT working, carbon counter and pseudo Ag/AgCl reference, were printed separately on a single substrate, using different printing techniques. Carbon and Ag/AgCl inks were manufactured in-house and applied to the fabrication of the counter and reference electrodes, respectively. A working electrode template (working electrode: 3 mm diameter and tracks: 35.6 mm × 2 mm) was designed using Silhouette Studio software and the pattern was transferred to a self-adhesive vinyl plastic sheet (70 µm ± 10% thickness) employing an electronic craft cutter (Silhouette Cameo, Silhouette America, Inc., Lindon, UT, USA). After the template (open mesh) was attached to the pre-treated PET sheet, the CNO/GRT ink was spread across it using a metal squeegee. The electrodes were cured in an oven for 15 min at 100 °C. The vinyl mask was then removed and an insulator layer was deposited to define the electrode working area of 0.0707 cm^2^, resulting in a 70 µm ± 5% thickness working electrode. The carbon/graphite counter and Ag/AgCl pseudo-reference electrodes were screen-printed using a DEK-248 semi-automatic screen-printer. All electrodes were used in a window of 4 weeks after the printing process. No further stability tests were performed.

## 4. Conclusions

Screen printing is currently a common strategy for the low-cost mass-production of disposable sensors. The novel CNO/GRT SPEs reported here show useful electrochemical performance and merit further investigation for specific applications, such as the reduction of oxygen and even carbon dioxide, as well as the development of biosensors. It appears reasonable to expect that, as strategies for the enhanced dispersion of CNOs are developed, the performance of the resulting SPEs will be further enhanced in the future.

## Figures and Tables

**Table 1:** Optimised ink formulation of the carbon nano-onion (CNO)/graphite (GRT) SPEs.

Material	Wt./Wt.%
Polyhydroxyethers	10
Di(propylene glycol) methyl ether	55
Poly(dimethylsiloxane-co-methylphenylsiloxane)	4
Carbon nano-onions	24
Graphite	7

**Table 2:** Chemical composition of the CNO/GRT SPEs from theoretical calculation and EDS analysis.

Element	Estimated Weight%	EDS Weight%
C	93.4	80.6
O	5.2	18.1
Si	1.4	1.3

**Table 3:** Elemental composition of p-CNOs, graphite and CNO/GRT SPEs from XPS analyses.

Sample	C (at. %)	O (at. %)	Si (at. %)
p-CNOs	93.7	5.6	0.8
Graphite	96.8	2.9	0.3
CNO/GRT SPEs	79.5	17.8	2.7

**Table 4:** Comparison of different electrode platforms modified with carbon (nano)materials applied for the sensing of DA.

Electrode	Linear Working Range (μM)	Dopamine (DA) LOD (µM)	Electrochemical Method	Reference
Graphene oxide nanoribbons/SPE	0.5–300.0	0.15	Differential pulse voltammetry	[60]
Reduced graphene oxide/TiO_2_ {001}/GCE	2.0–60.0	6.00	Differential pulse voltammetry	[61]
Magnetic multi-walled carbon nanotubes/SPE	5.0–180.0	0.43	Square wave voltammetry	[62]
Nanodiamonds/SPE	2.0–100.0	0.57	Differential pulse voltammetry	[63]
3D porous graphene oxide-gold nanoparticle/ITO	0.1–30.0	1.28	Cyclic Voltammetry	[64]
Nitrogen-doped reduced graphene oxides (150-2)/GCE	3.0–70.0	1.50	Differential pulse voltammetry	[65]
CNO/GRT SPE	10.0–99.9	0.92	Cyclic Voltammetry	This work

## Supplementary Materials

The following are available online, Figure S1: Cyclic voltammograms of CNO/GRT SPE containing different weight percentages of CNO particles: (**a**) Optimised ink formulation, (**b**) 70 wt.% CNO, (**c**) 60 wt.% CNO and (**d**) 50 wt.% CNO. The inset graph shows the c and d voltammograms, Figure S2: SEM image of the CNO/GRT SPE surface at 1400× as magnification. Graphite flakes and spherical CNO aggregates are highlighted through white and blue circles, respectively, Figure S3: High-resolution C 1s XPS spectra of (**A**) p-CNOs and (**B**) graphite, including peak deconvolution. The experimental and fitting curves are shown in black and red, respectively, Figure S4: High-resolution O 1s XPS spectra of (**A**) p-CNOs and (**B**) graphite, including peak deconvolution. The experimental and fitting curves are shown in black and red, respectively, Figure S5: Cyclic voltammograms of the commercial GRT SPE in 1 mM FcMeOH/PBS pH 7.4 at different scan rates (10, 20, 50, 70, 100, 150, and 200 mV·s^−1^). Inset graph: Ipa and Ipc versus square root of scan rate, *υ^1/2^,* Figure S6: (**A**) Cyclic voltammograms of commercial GRT SPE in PBS pH 7.4 after subsequent additions of dopamine, in the range 10.0–99.9 µM. (**B**) Calibration plot of the anodic peak current as a function of the dopamine concentration (N = 3). Table S1: Chemical states, positions and relative area percentages of the deconvoluted C 1s peaks of p-CNOs, graphite and CNO/GRT SPE from XPS analyses, Table S2: Chemical states, positions and relative area percentages of the deconvoluted O 1s peaks of p-CNOs, graphite and CNO/GRT SPE from XPS analyses, Table S3: Chemical states, positions and relative area percentages of the deconvoluted Si 2p peak of CNO/GRT SPE from XPS analyses.
