# Supplementary material for: Electrochemical Properties of Screen-Printed Carbon Nano-Onion Electrodes
Source: Molecules. 2020 Aug 26;25(17):3884. doi: 10.3390/molecules25173884 (PMC7503887; doi:10.3390/molecules25173884)
Supplement: Supplementary file 1 [file molecules-25-03884-s001.pdf]

Supplementary Materials

Electrochemical Properties of Screen-Printed Carbon Nano-Onion Electrodes

Loanda R. Cumba [1,*], Adalberto Camisasca [1], Silvia Giordani [1,*] and Robert J. Forster [1,2]

[1] School of Chemical Sciences, National Centre for Sensor Research, Dublin City University, Dublin 9, Ireland; Adalberto.camisasca@dcu.ie (A.C.); robert.forster@dcu.ie (R.J.F.)
[2] FutureNeuro SFI Research Centre, Dublin, Ireland
* Correspondence: loanda.cumba@dcu.ie (L.R.C.); Silvia.Giordani@dcu.ie (S.G.)

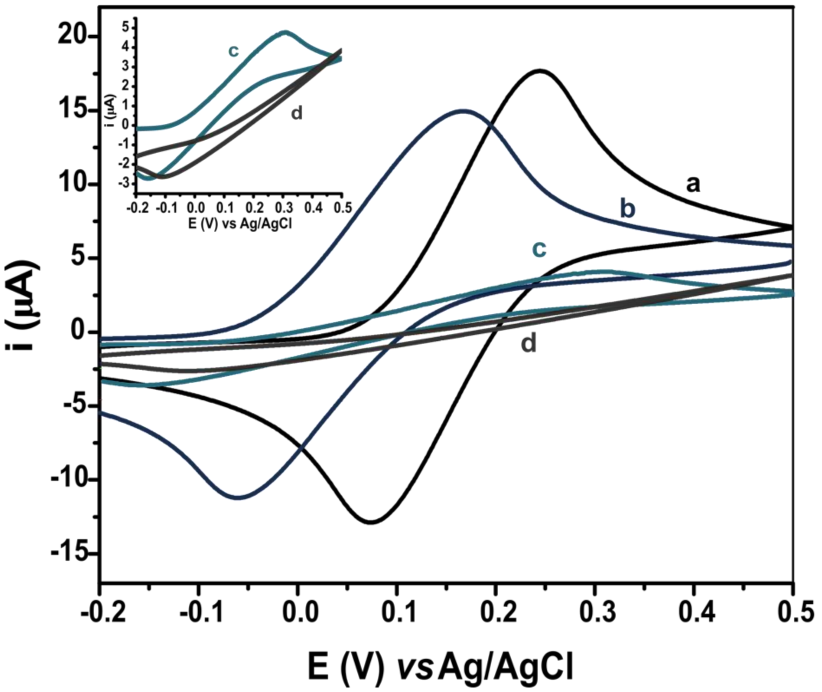

Figure S1. Cyclic voltammograms of CNO/GRT SPE containing different weight percentages of CNO particles: (**a**) Optimised ink formulation, (**b**) 70 wt.% CNO, (c) 60 wt.% CNO and (d) 50 wt.% CNO. The inset graph shows the c and d voltammograms.

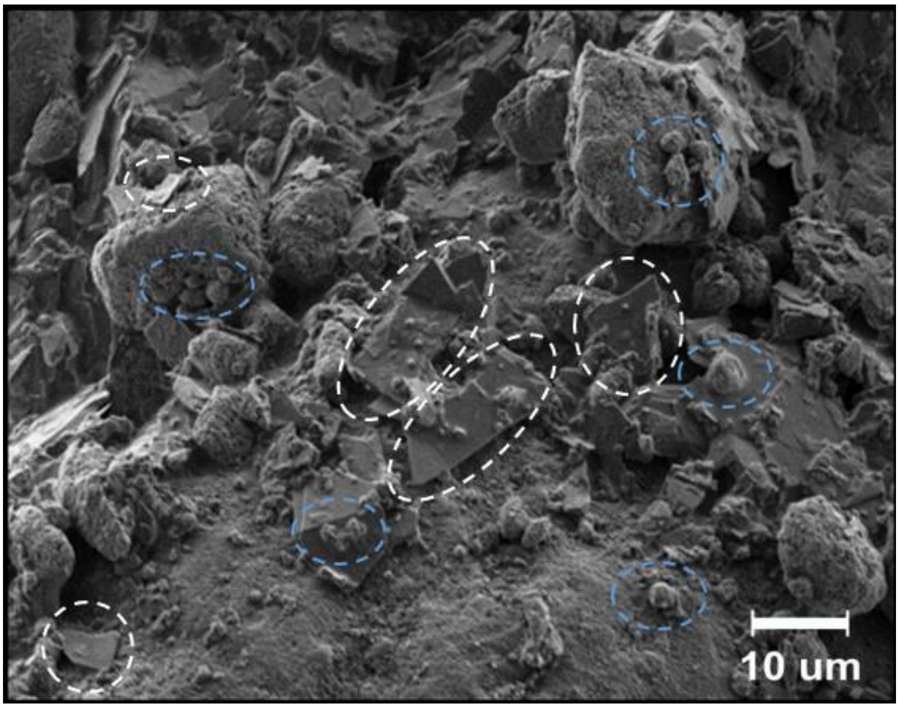

Figure S2: SEM image of the CNO/GRT SPE surface at 1,400x as magnification. Graphite flakes and spherical CNO aggregates are highlighted through white and blue circles, respectively.

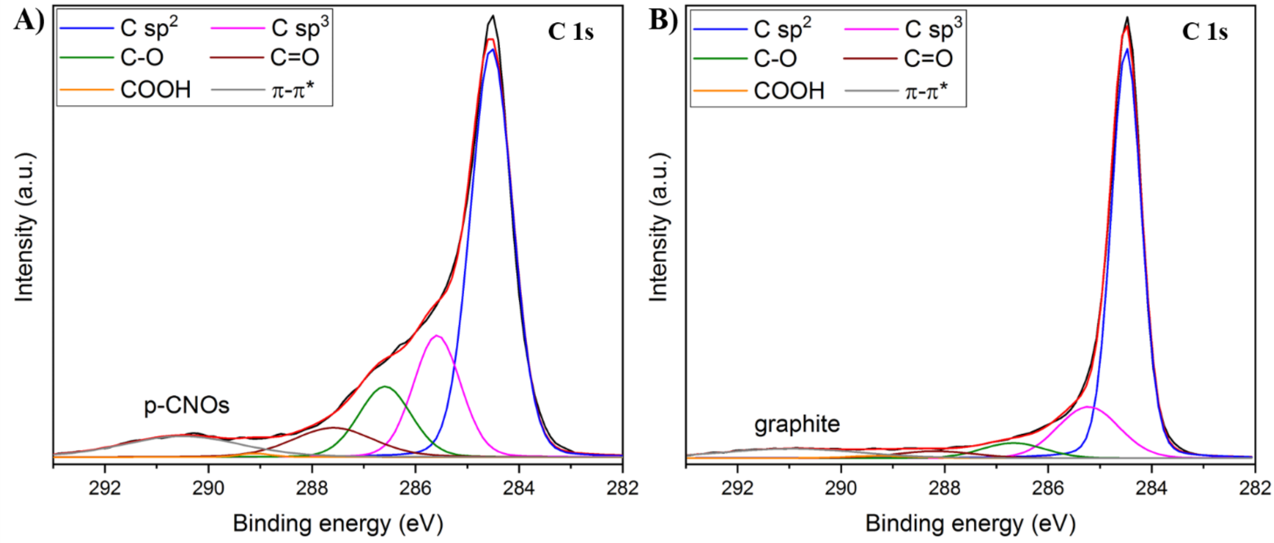

Figure S3. High-resolution C 1s XPS spectra of **A**) p-CNOs and **B**) graphite, including peak deconvolution. The experimental and fitting curves are shown in black and red, respectively.

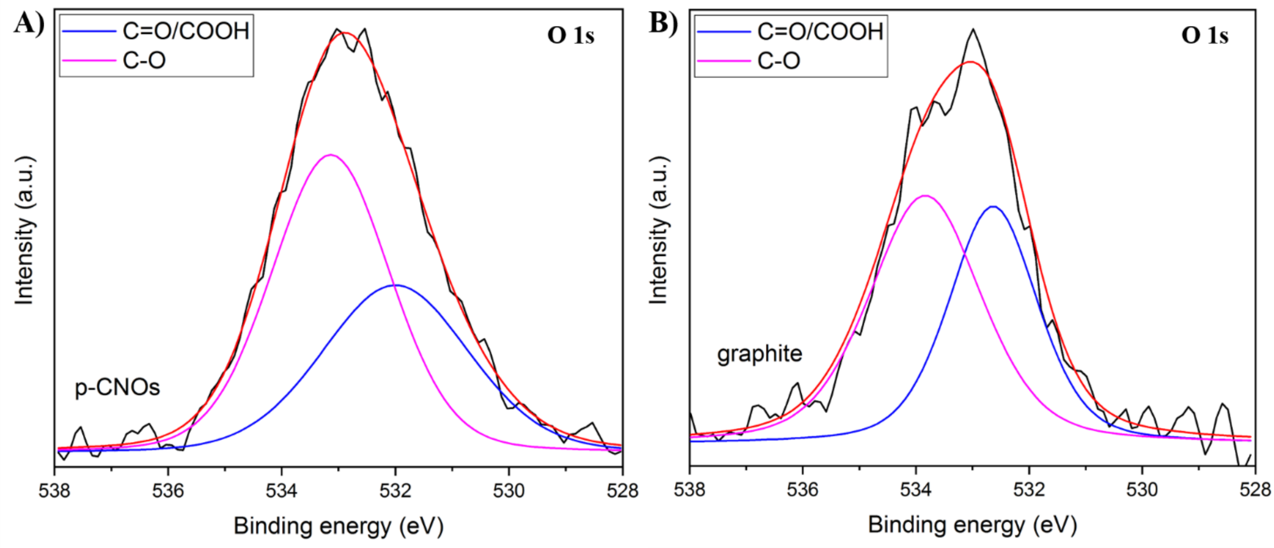

Figure S4. High-resolution O 1s XPS spectra of A) p-CNOs and B) graphite, including peak deconvolution. The experimental and fitting curves are shown in black and red, respectively.

Table S1. Chemical states, positions and relative area percentages of the deconvoluted C 1s peaks of p-CNOs, graphite and CNO/GRT SPE from XPS analyses.

**Sample**	**C-C sp²** (eV)	**C-C sp³** (eV)	**C-O** (eV)	**C=O** (eV)	**COOH** (eV)	**π-π*** (eV)
p-CNOs	284.5 (54.2 %)	285.6 (17.9 %)	286.6 (11.6 %)	287.6 (7.8 %)	289.1 (0.4 %)	290.5 (8.1 %)
Graphite	284.5 (68.6 %)	285.2 (16.4 %)	286.7 (4.9 %)	288.2 (2.8 %)	289.3 (0.7%)	291.0 (6.6 %)
CNO/GRT SPE	284.5 (53.8 %)	285.6 (15.9 %)	286.7 (20.0 %)	288.1 (5.6 %)	289.9 (1.5%)	291.3 (3.2 %)

Table S2. Chemical states, positions and relative area percentages of the deconvoluted O 1s peaks of p-CNOs, graphite and CNO/GRT SPE from XPS analyses.

**Sample**	**C=O/COOH** (eV)	**C-O** (eV)	**O-H** (eV)
p-CNOs	532.0 (41.0 %)	533.1 (59.0 %)	-
Graphite	532.6 (43.1 %)	533.8 (56.9 %)	-
CNO/GRT SPE	532.3 (20.7 %)	533.3 (57.4 %)	535.3 (21.9 %)

Table S3. Chemical states, positions and relative area percentages of the deconvoluted Si 2p peak of CNO/GRT SPE from XPS analyses.

Sample	Si-O-Si (eV)	SiO_2 (eV)
CNO/GRT SPE	102.3 (88.1 %)	104.4 (11.9 %)

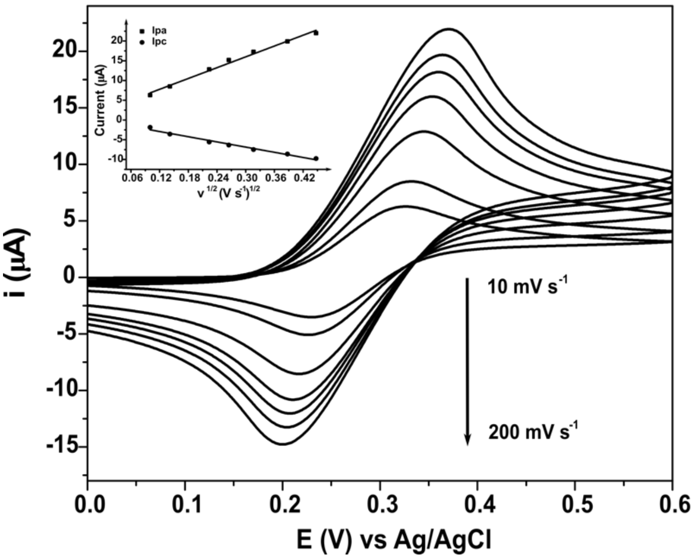

Figure S5. Cyclic voltammograms of the commercial GRT SPE in 1 mM FcMeOH/PBS pH 7.4 at different scan rates (10, 20, 50, 70, 100, 150, and 200 mV·s^{-1}). Inset graph: I_{pa} and I_{pc} versus square root of scan rate, $\upsilon^{1/2}$.

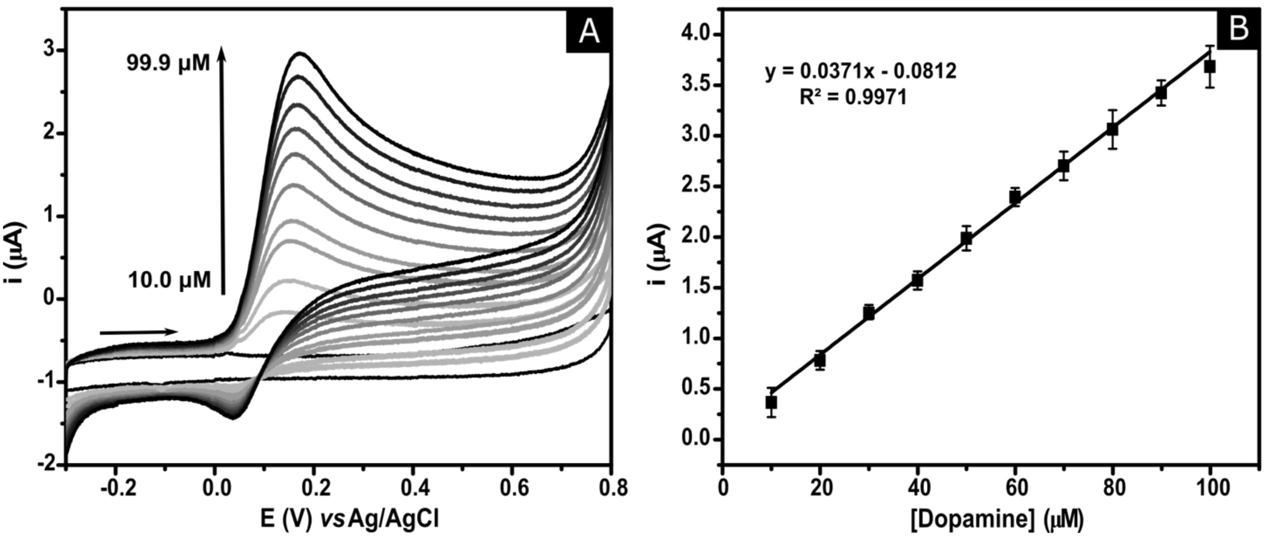

Figure S6. (**A**) Cyclic voltammograms of commercial GRT SPE in PBS pH 7.4 after subsequent additions of dopamine, in the range 10.0-99.9 μM. (**B**) Calibration plot of the anodic peak current as a function of the dopamine concentration (N = 3).
